# The effect of sport event brand perception on revisit intention: the mediating roles of experiential satisfaction and emotional connection

**DOI:** 10.3389/fspor.2026.1884993

**Published:** 2026-08-07

**Authors:** Lei Ma, JoonYoung Han

**Affiliations:** 1 Department of Physical Education, Hexi University, Zhangye, Gansu, China; 2 Department of Physical Education, Yeungnam University, Gyeongsan, Republic of Korea

**Keywords:** brand perception, emotional connection, experiential satisfaction, revisit intention, sport event

## Abstract

**Background:**

In the increasingly competitive sport event market, brand perception has emerged as a key factor influencing individuals’ behavioral intentions. However, the internal mechanism through which sport event brand perception affects revisit intention remains insufficiently understood, particularly with regard to the roles of experiential satisfaction and emotional connection.

**Methods:**

This study examined how sport event brand perception influences individuals’ revisit intention through experiential satisfaction and emotional connection. Individuals involved in the Beijing Marathon event were recruited for a questionnaire survey, and 528 valid responses were obtained. The data were analyzed using SPSS and AMOS. Reliability and validity were assessed, and structural equation modeling was employed to test the hypothesized relationships and mediation effects.

**Results:**

Sport event brand perception had significant positive effects on experiential satisfaction, emotional connection, and revisit intention. Experiential satisfaction fully mediated the relationship between brand perception and revisit intention. In contrast, the mediating effect of emotional connection was not statistically significant.

**Conclusion:**

This study identifies the differentiated mechanisms through which sport event brand perception influences individuals’ revisit intention, highlighting the dominant role of experiential satisfaction over emotional connection in the Beijing Marathon context. The findings provide practical implications for sport event organizers seeking to enhance individuals’ intention to revisit the event by strengthening brand perception and systematically optimizing event experiences.

## 1 Introduction

Large-scale mass-participation sports events have become important platforms for city branding, public engagement, and sport consumption (1, 2). Among these events, marathons are particularly representative because participation involves more than the sporting activity itself. Individuals engaging with these events also evaluate event services, destination attributes, and social interactions throughout the event experience. As competition among host cities and marathon events continues to intensify, attracting individuals to engage with these events is no longer sufficient. Event organizers must also encourage repeat participation to ensure long-term sustainability. In this context, sport event brand perception has emerged as an important factor influencing individuals’ behavior. Sport event brand perception refers to individuals’ overall evaluation of event quality, symbolic meaning, and social influence (3). Previous studies have shown that favorable brand perception enhances event attractiveness, improves individuals’ evaluations, and increases revisit intention (4, 5). However, the psychological process through which brand perception translates into revisit intention remains insufficiently understood, particularly in high-involvement endurance event settings.

The Stimulus–Organism–Response (SOR) framework provides a useful theoretical basis for explaining this process (6). Within this framework, sport event brand perception functions as an external stimulus that shapes individuals’ internal psychological states and subsequently influences behavioral responses, including revisit intention (7, 8). Existing studies have largely confirmed the direct relationship between brand perception and revisit intention. Yet the internal mechanism linking these variables remains unclear. More specifically, it is still uncertain whether brand perception influences revisit intention mainly through individuals’ functional evaluation of the event experience or through a deeper emotional bond with the event.

Experiential satisfaction and emotional connection represent two distinct psychological mechanisms that may explain this relationship. Experiential satisfaction reflects individuals’ cognitive evaluation of their overall event experience and indicates whether the event meets or exceeds expectations. Emotional connection refers to the affective bond individuals develop toward an event (9, 10). Prior studies suggest that positive brand-related experiences can strengthen both satisfaction and emotional attachment, which in turn influence future behavioral intentions. However, sport event research has rarely examined these two mechanisms simultaneously. Their relative importance in shaping revisit intention therefore remains unclear. Distinguishing between these pathways is important because it helps explain whether revisit intention is driven primarily by functional evaluation or affective attachment.

This issue becomes more important when research context is considered. Much of the sport branding literature has focused on spectator sports, where fandom, symbolic meaning, and emotional attachment strongly influence consumer behavior (11). Mass-participation endurance events differ in important ways. Compared with spectator sports, mass-participation events require a higher level of involvement from individuals engaging with the event, whether through athletic participation or other forms of on-site engagement. These individuals continuously evaluate event organization, safety management, and operational efficiency throughout the event experience (12). Because of these characteristics, functional event delivery may play a more central role than emotional attachment in shaping revisit intention. This suggests that the mechanism linking brand perception and revisit intention may differ between purely spectator sport and mass-participation event contexts.

This study focuses on the Beijing Marathon, one of the most established and influential marathon events in China. Since its inception in 1981, the Beijing Marathon has contributed to sport participation, sport tourism, and urban branding (13, 14). Prior research also suggests that large-scale sports events can generate broader economic and tourism benefits for urban development (15). However, as competition among marathon events intensifies, sustaining long-term engagement with the event has become increasingly important, making it necessary to better understand the psychological factors underlying revisit intention.

This study examines how sport event brand perception influences revisit intention in the Beijing Marathon context, with particular attention to the mediating roles of experiential satisfaction and emotional connection. The findings contribute to the sport event branding literature in three ways. First, this study extends prior research by moving beyond direct relationships and examining the psychological mechanisms linking brand perception and revisit intention within the SOR framework. Second, by comparing the mediating effects of experiential satisfaction and emotional connection, the study provides a deeper understanding of their relative importance in shaping revisit intention in endurance events. Third, although previous studies have primarily emphasized emotional attachment as a key driver of loyalty in spectator sport contexts, the present findings suggest that this pattern may differ in mass-participation sports events. In these high-involvement event settings, experiential satisfaction may play a more prominent role than emotional connection in translating brand perception into revisit intention. These findings also offer practical implications for event organizers seeking to strengthen brand value, enhance event experiences, and encourage long-term engagement.

## 2 Theoretical foundations and research hypotheses

### 2.1 Sport event brand perception

Mass-participation marathons have evolved beyond purely athletic competitions and increasingly reflect the characteristics of event tourism and experiential consumption (4). Engagement with a marathon event involves more than completing the race itself; individuals also interact with the host city, event services, social relationships, and the overall event atmosphere. As a form of event tourism, marathon events can also generate broader tourism and economic benefits for host destinations, contributing to regional development and inclusive economic growth (16). In this sense, individuals derive value not only from the sporting activity but also from destination-related experiences shaped by urban settings, cultural meanings, and event narratives. Prior research on experiential consumption suggests that in highly involving contexts, consumers evaluate experiences based on both functional outcomes and memorable cognitive, emotional, and social responses (17). Accordingly, marathons can be understood as highly experience-driven events, where individuals’ evaluations are influenced not only by service quality but also by the symbolic meanings attached to the event.

Within this context, sport event brand perception becomes an important reference point for individuals’ expectations and evaluations. Drawing upon Brand Equity Theory to conceptualize the external stimulus within the SOR framework, sport event brand perception is defined here as how individuals recognize, understand, and evaluate a brand (18). In sport event settings, brand perception involves more than simple awareness of an event name. It reflects individuals’ overall evaluation of the event's image, organizational professionalism, and symbolic value. Previous studies suggest that sport event brand perception can be understood through three dimensions: brand awareness, brand image, and brand trust. Brand awareness refers to the extent to which individuals recognize and recall the event (19). Brand image reflects symbolic associations and perceived experiential value (20). Brand trust represents confidence in the organizer's capability and service reliability (21).

Although these dimensions are often examined separately, individuals engaging with sport events rarely evaluate them in isolation. In high-involvement events such as marathons, individuals tend to form an overall impression of the event brand rather than making fully independent judgments about awareness, image, and trust (22). Awareness increases familiarity, image shapes meaning, and trust reduces uncertainty. Together, these dimensions contribute to individuals’ overall perception of the event brand. Therefore, this study primarily conceptualizes sport event brand perception as a higher-order construct reflected by brand awareness, brand image, and brand trust, consistent with recent sport branding research emphasizing the multidimensional nature of brand-related consumer evaluations (23). At the same time, because these first-order dimensions may contribute differently to individuals’ psychological responses and behavioral intentions, their individual effects are also examined as supplementary analyses.

### 2.2 Psychological mediating mechanisms: experiential satisfaction and emotional connection

Within the overarching Stimulus–Organism–Response (SOR) framework, this study integrates Affective Events Theory to explicate individuals’ internal psychological states (24). Because this theory posits that external events trigger simultaneous cognitive appraisals and affective reactions, the current model proposes two parallel mediating mechanisms: experiential satisfaction as the cognitive evaluation process and emotional connection as the affective response. Experiential satisfaction reflects individuals’ cognitive evaluation of their event experience, whereas emotional connection captures the affective bond developed through meaningful experiences and interactions with the event. Examining both mechanisms simultaneously helps explain whether revisit intention in marathon events is driven more strongly by functional evaluation or emotional attachment.

Experiential satisfaction refers to individuals’ overall evaluation of their actual event experience relative to prior expectations (25). In sport event research, it is widely regarded as an important indicator of service performance and experiential quality because it reflects individuals’ integrated judgments of event organization, service quality, race support, and the on-site environment (26). When event delivery meets or exceeds expectations, individuals are more likely to report higher satisfaction, which strengthens favorable attitudes and increases revisit intention (27). In mass-participation endurance events, experiential satisfaction may be particularly important because individuals directly experience the operational quality of the event throughout the race and their time at the event venue. As a result, satisfaction provides a functional pathway through which brand perception may influence future behavioral intention.

Emotional connection reflects the affective bond individuals develop with a sport event through repeated interaction and meaningful experiences. It is characterized by emotional attachment, psychological closeness, and a sense of belonging (28). Although emotional connection is related to brand identification, the two constructs are conceptually distinct. Brand identification mainly reflects cognitive alignment between an individual and a brand, meaning that the brand becomes part of one's self-concept. Emotional connection extends beyond this cognitive alignment by emphasizing affective attachment and emotional bonding formed through participation experiences. Grounded in Attachment Theory, which elucidates how affective bonds translate into long-term behavioral responses, strong emotional connections can reinforce symbolic meaning, increase revisit intention, and ultimately strengthen individuals’ intention to revisit the event (29, 30). Accordingly, emotional connection represents the affective pathway through which sport event brand perception may shape revisit intention.

### 2.3 Research hypotheses

Building on the theoretical foundations discussed above, this study develops hypotheses based on the Stimulus–Organism–Response (SOR) framework to examine the relationships among sport event brand perception, experiential satisfaction, emotional connection, and revisit intention. In tourism and service contexts, favorable brand perceptions often reduce uncertainty and perceived risk, making consumers more willing to revisit or repurchase (31). This effect becomes more salient when participation involves considerable effort, cost, and commitment, as individuals tend to rely more heavily on brand-related judgments when making decisions (32).

This logic is particularly relevant in marathon events. Engaging with a large-scale marathon event often requires considerable time, financial investment, and personal involvement, regardless of the specific form of participation (33). Under such conditions, individuals are more likely to rely on their overall perception of the event brand when deciding whether to engage with the event again. A strong sport event brand signals reliable organization, service quality, and event prestige, which strengthens trust and increases willingness to return (34). Therefore, the following hypothesis is proposed:

H1: Sport event brand perception positively influences individuals’ revisit intention.
H1a: Brand awareness positively influences individuals’ revisit intention.
H1b: Brand image positively influences individuals’ revisit intention.
H1c: Brand trust positively influences individuals’ revisit intention.

Experiential satisfaction is generally understood as an overall post-experience evaluation that links cognitive judgment to later behavioral intention (35). Previous studies suggest that individuals’ pre-experience brand perception shapes their expectations, and these expectations influence how they interpret the event and evaluate satisfaction during the actual experience (36). In tourism research, Stylos et al. showed that a favorable destination image contributes to a more positive holistic evaluation and strengthens revisit intention, indicating that prior image-related perceptions can shape how individuals evaluate subsequent experiences (37). Similarly, Lien et al. found that a positive brand image enhances trust and perceived value, which in turn increase consumers’ purchase intentions, suggesting that favorable brand-related perceptions can improve subsequent evaluations of the service encounter and related behavioral responses (38). Sport event studies also show that perceptions of professionalism and credibility can enhance individuals’ experiential satisfaction with event organization, service delivery, and the on-site environment (27). Therefore, the following hypotheses are proposed:

H2: Sport event brand perception positively influences individuals’ experiential satisfaction.
H2a: Brand awareness positively influences individuals’ experiential satisfaction.
H2b: Brand image positively influences individuals’ experiential satisfaction.
H2c: Brand trust positively influences individuals’ experiential satisfaction.

Emotional connection refers to the affective dependence and psychological identification that individuals develop with a focal target through long-term interaction and accumulated experience (39). Consumer and brand research identifies emotional connection as a core psychological mechanism that supports long-term relationship maintenance and repeated behavior. Matthews, Son, and Watchravesringkan argued that brand image and brand trust provide an important basis for emotional connection and that consumers who hold clearer and more favorable brand perceptions are more likely to develop emotional attachment (40).

Related arguments also appear in experience-centered consumption and service research. Studies in the restaurant industry, for example, have shown that brand personality shapes customer cognition, emotional relationships, and brand image formation, while brand awareness and brand image strengthen emotional connection with the brand (41). Rinaldi, Millanyani, and Trianasari further reported in the context of social media marketing that stronger brand-related perceptions are associated with stronger emotional engagement and affective responses (42). In sport events, brands often carry symbolic meanings related to city image, collective identity, and personal achievement. Strong brand perception may therefore help individuals form a stable emotional relationship with the event (11). Given the close association between large-scale marathon events and destination promotion, emotional relationships may also be reinforced by the broader symbolic value conveyed by the event brand rather than by destination attributes alone. Therefore, the following hypotheses are proposed:

H3: Sport event brand perception positively influences individuals’ emotional connection.
H3a: Brand awareness positively influences individuals’ emotional connection.
H3b: Brand image positively influences individuals’ emotional connection.
H3c: Brand trust positively influences individuals’ emotional connection.

Experiential satisfaction may also mediate the relationship between sport event brand perception and revisit intention. Following the event experience, experiential satisfaction reflects individuals’ subjective judgment of how well the event experience matches prior expectations and thus captures a key process through which cognitive evaluation becomes behavioral intention (43). Consumer and service research has long treated experiential satisfaction as a representative mediator that converts external stimuli into later behavioral responses (44). When individuals hold favorable perceptions of an event brand, they tend to form clearer and more positive expectations. These expectations then lead them to interpret event organization, service quality, and the overall atmosphere more favorably during the experience. As a result, individuals evaluate the event more positively and report higher satisfaction (45).

Empirical studies in sport and tourism contexts also support this mechanism. Liu et al. found that, in a sports event context, event quality significantly enhanced tourists’ satisfaction and indirectly increased revisit intention through satisfaction (46). Li et al. likewise showed in tourism research that experiential satisfaction serves as a key mediating mechanism between cognitive evaluation and revisit intention (47). Together, these findings indicate that experiential satisfaction is likely to be a critical pathway through which sport event brand perception translates into revisit intention. Therefore, the following hypothesis is proposed:

H4: Experiential satisfaction mediates the relationship between sport event brand perception and individuals’ revisit intention.

Emotional connection may constitute another important mediating mechanism. Emotional connection refers to the affective attachment and psychological identification that gradually develop through repeated interaction and continuing experience, and researchers widely regard it as a key affective mechanism underlying long-term relationship maintenance and repeated behavior (30). In brand research, emotional connection captures not only functional evaluation but also emotional immersion and symbolic meaning. When individuals form a clear and stable perception of a sport event brand, they are more likely to view the event as emotionally meaningful and symbolically valuable, which can foster sustained emotional attachment at the psychological level (29). This process suggests that brand perception may also influence behavioral intention through an affective pathway.

Empirical studies support this argument. In the context of sport tourism, Brown, Smith, and Assaker found that individuals’ positive evaluations of a sports event significantly strengthen their emotional attachment to the host destination, which in turn acts as a critical affective mediator driving their intentions to revisit (48). Tourism research has also shown that affective connection functions as a key emotional foundation in the process through which cognitive and affective destination images influence satisfaction and revisit intention, with stronger emotional connection associated with significantly higher satisfaction (49). These findings indicate that emotional connection may serve as a core affective mechanism linking sport event brand perception and revisit intention. Its mediating role therefore merits empirical examination in the sport event context. Therefore, the following hypothesis is proposed:

H5: Emotional connection mediates the relationship between sport event brand perception and individuals’ revisit intention.

## 3 Materials and methods

### 3.1 Participants and data collection

This study surveyed individuals involved in the 2024 Beijing Marathon event. Founded in 1981, the Beijing Marathon is one of the oldest marathon races in China and remains one of the country's most influential sport events. Official statistics show that 182,949 people applied for the 2024 race, with participants and visitors coming from 43 countries and regions (50).

Convenience sampling was employed to collect the data. An online questionnaire was distributed via Wenjuanxing, a widely used survey platform in China, to individuals who engaged in the 2024 Beijing Marathon event, including race participants and on-site attendees. The purpose of this study was to examine overall perceptions of the event brand and the psychological mechanisms underlying revisit intention, rather than to compare different participant roles. Accordingly, all respondents were analyzed as a single analytical group. Potential differences between race participants and on-site attendees were beyond the scope of the present study and are recommended for future investigation using multi-group structural equation modeling. Data collection was conducted shortly after the event to ensure that respondents could evaluate their experiences while these remained fresh. Because the questionnaire was administered only in Chinese, the sample was restricted to Chinese-speaking participants. A total of 723 responses were received. After removing incomplete questionnaires and responses exhibiting clear response bias, 528 valid questionnaires were retained, yielding an effective response rate of 73.0%. The final sample size exceeded the recommended minimum for structural equation modeling, which is generally considered adequate with at least 200 observations for reliable parameter estimation (51). Therefore, the sample size was considered sufficient for the subsequent analyses. Demographic characteristics of the respondents are presented in Table 1.

**Table 1:** Demographic characteristics of the individuals involved in the Beijing Marathon (*N* = 528).

Characteristic	Category	*n*	%
Gender	Male	268	50.8
	Female	260	49.2
Age	Under 18	29	5.5
	18–29	163	30.9
	30–39	123	23.3
	40–49	118	22.3
	50–59	53	10
	60 or above	42	8
Education level	Middle school graduate or below	32	6
	High school graduate	42	8
	Junior college graduate	161	30.5
	Bachelor's degree	243	46
	Master's degree or above	50	9.5
Monthly income	CNY 3,000 or below	71	13.4
	CNY 3,001–5,000	94	17.8
	CNY 5,001–8,000	195	36.9
	CNY 8,001–12,000	119	22.5
	Above CNY 12,000	49	9.4
Occupation	Government or public institution staff	51	9.7
	Corporate management or professional technical staff	118	22.4
	Company employee	58	11
	Commerce or service worker	37	7
	Self-employed	122	23.1
	Student	82	15.5
	Retired	29	5.5
	Other	31	5.8
Total		528	100

Monthly income is reported in Chinese yuan (CNY).

Percentages may not sum to 100 due to rounding.

### 3.2 Measures

This study measured four key constructs: sport event brand perception, experiential satisfaction, emotional connection, and revisit intention. Following the conceptual framework of the study, we treated brand perception as a higher-order construct and operationalized it in the sports event context based on Brand Equity Theory (18, 22). To enhance the applicability of the measurement scales across the study sample, the measurement items were adapted to capture the overall event experience shared by individuals involved in the event while preserving the conceptual meaning of the original constructs. We measured brand perception through three dimensions: brand awareness, brand image, and brand trust. The items were adapted from validated scales used in previous studies on branding and sport events, including Bańbuła, Martínez, Alguacil, and Calabuig, and Chaudhuri and Holbrook. We adapted and reworded several items to enhance their applicability across different forms of involvement in the Beijing Marathon event while maintaining consistency with the original validated scales (8, 19, 21).

We measured experiential satisfaction using the framework developed and validated by Jin, Lee, and Lee in sport event research. The items captured three core experiential components: event quality, service level, and event environment (52). These components represent the functional and experiential aspects of the event that are applicable across different forms of event involvement. We assessed emotional connection using the approach proposed by Alonso and Montoro, which captures the emotional relationship between individuals and the event through emotional dependence, brand identification, and social connection (53). We measured revisit intention with items adapted from Hasan et al., focusing on individuals’ likelihood of attending the same sports event again in the future (54).

Before the main data collection, a pilot survey was conducted to assess item clarity, response consistency, and the psychometric properties of the measurement scales. The pilot questionnaire was administered online to individuals with prior Beijing Marathon experience, including both race participants and event attendees, yielding 323 valid responses from an initial pool of 368. To avoid sample overlap, these respondents were excluded from the main survey. Reliability and exploratory factor analyses demonstrated satisfactory measurement properties, with Cronbach's *α* values exceeding.70, KMO values above.90, significant Bartlett's tests of sphericity (*p* < .001), and factor loadings above.70. Based on pilot feedback and expert consultation, several items were refined to improve wording clarity and contextual relevance to the Beijing Marathon setting. All items in the final questionnaire were measured using a 5-point Likert scale ranging from 1 (strongly disagree) to 5 (strongly agree), with the complete measurement items provided in Appendix A. Given that the Beijing Marathon represents a large-scale participatory sports event rather than a purely competitive race, the measurement instruments were adapted to capture individuals’ holistic evaluations of the overall event experience, including event organization, service delivery, event atmosphere, and interactions within the event environment. Accordingly, certain brand image items were intended to capture the broader symbolic value conveyed by the event brand rather than purely destination-related perceptions. This approach reflects the multidimensional nature of city-based marathon events, which function not only as sporting competitions but also as platforms for social engagement and destination branding.

### 3.3 Data analysis

All statistical analyses were performed using SPSS and AMOS. First, descriptive statistics were computed to examine the demographic characteristics of the sample, and the reliability and validity of the measurement instruments were assessed. Second, after data screening and cleaning, confirmatory factor analysis (CFA) was conducted to evaluate the measurement model fit and the factor loadings of individual items. Finally, structural equation modeling (SEM) was used to test the hypothesized relationships among the study variables and examine the proposed mediation effects.

## 4 Results

### 4.1 Reliability and validity of the measurement model

To account for the self-reported nature of our data, which captured respondents’ subjective perceptions, common method bias was assessed using the common latent factor approach in AMOS (55). The assessment was conducted using responses from the full sample described above. A common latent factor was added to the measurement model, with all measurement items loading simultaneously on their respective theoretical constructs and the common latent factor. The comparison of standardized factor loadings before and after the inclusion of the common latent factor indicated no substantial changes in item loadings. Following the recommended criterion, the differences in standardized factor loadings were below.20, suggesting that common method bias was unlikely to pose a serious threat to the validity of the findings.

Confirmatory factor analysis was conducted using AMOS to assess the measurement model. As shown in Table 2, all constructs demonstrated satisfactory reliability, with Cronbach's alpha and composite reliability exceeding.70. AVE values were also above the recommended threshold of.50, indicating adequate convergent validity (51). Model fit indices further suggested that the measurement model fit the data well. Given that sport event brand perception, experiential satisfaction, and emotional connection were specified as higher-order constructs, a second-order confirmatory factor analysis was performed to examine the hierarchical measurement structure. All first-order dimensions loaded significantly on their respective higher-order constructs, with standardized loadings exceeding.70 (*p* < .001), supporting the validity of the higher-order measurement structure.

**Table 2:** Standardized factor loadings, composite reliability, and convergent validity of the constructs.

Construct	Item	Standardized loading	CR	AVE	Cronbach's *α*
Brand perception	Brand awareness 1	.877	.890	.729	.888
	Brand awareness 2	.826			
	Brand awareness 3	.857			
	Brand image 1	.884	.928	.762	.927
	Brand image 2	.879			
	Brand image 3	.885			
	Brand image 4	.843			
	Brand trust 1	.840	.889	.727	.889
	Brand trust 2	.860			
	Brand trust 3	.857			
Experiential satisfaction	Event quality 1	.847	.904	.701	.902
	Event quality 2	.862			
	Event quality 3	.850			
	Event quality 4	.789			
	Service level 1	.825	.913	.723	.912
	Service level 2	.847			
	Service level 3	.875			
	Service level 4	.853			
	Event environment 1	.816	.891	.672	.891
	Event environment 2	.831			
	Event environment 3	.816			
	Event environment 4	.817			
Emotional connection	Emotional dependence 1	.839	.869	.689	.869
	Emotional dependence 2	.825			
	Emotional dependence 3	.826			
	Brand identification 1	.901	.899	.748	.898
	Brand identification 2	.884			
	Brand identification 3	.807			
	Social connection 1	.856	.898	.745	.898
	Social connection 2	.864			
	Social connection 3	.869			
Revisit intention	Revisit intention 1	.869	.918	.737	.918
	Revisit intention 2	.844			
	Revisit intention 3	.857			
	Revisit intention 4	.863			

Model fit indices: *χ*^2^/*df* = 1.455, RMSEA = .029, GFI = .919, SRMR = .058, NFI = .951, IFI = .984, and PGFI = .795.

CR, composite reliability; AVE, average variance extracted.

### 4.2 Discriminant validity assessment

Discriminant validity was assessed using the heterotrait–monotrait (HTMT) ratio of correlations, which is widely recommended as a more rigorous criterion than traditional approaches for assessing discriminant validity in structural equation modeling (56). As shown in Table 3, HTMT values were calculated for all first-order constructs underlying the higher-order constructs. All HTMT values were below the conservative threshold of.85, with the highest value being.796. These results indicate that the constructs demonstrate satisfactory discriminant validity.

**Table 3:** Discriminant validity based on HTMT (Heterotrait–Monotrait ratio).

Construct	BA	BI	BT	EQ	SL	EE	ED	BID	SC	RI
BA	–									
BI	.769	–								
BT	.751	.766	–							
EQ	.582	.594	.580	–						
SL	.750	.765	.746	.595	–					
EE	.721	.735	.717	.572	.737	–				
ED	.681	.694	.678	.526	.677	.651	–			
BID	.676	.689	.673	.522	.672	.646	.717	–		
SC	.607	.619	.604	.469	.604	.580	.644	.639	–	
RI	.780	.796	.776	.611	.787	.756	.748	.742	.667	–

Values in the table represent heterotrait–monotrait ratios (HTMT).

BA, brand awareness; BI, brand image; BT, brand trust; EQ, event quality; SL, service level; EE, event environment; ED, emotional dependence; BID, brand identification; SC, social connection; RI, revisit intention.

### 4.3 Direct effects of brand perception on revisit intention

As shown in Table 4, all three dimensions of sport event brand perception had significant positive effects on revisit intention. Specifically, brand awareness significantly predicted revisit intention (*β* = .267, *p* < .001), brand image also had a significant positive effect and showed the largest standardized coefficient among the three dimensions (*β* = .398, *p* < .001), and brand trust likewise exerted a significant positive effect on revisit intention (*β* = .242, *p* < .001). Overall, these findings demonstrate that all three dimensions of sport event brand perception significantly enhanced individuals’ revisit intention, thereby supporting the proposed direct relationship between sport event brand perception and revisit intention.

**Table 4:** Direct effects of the dimensions of brand perception on revisit intention.

Path	*B*	*β*	SE	CR
Brand awareness → Revisit intention	.233	.267	.057	4.074*
Brand image → Revisit intention	.368	.398	.063	5.854*
Brand trust → Revisit intention	.212	.242	.049	4.317*

Model fit indices: *χ*^2^/*df* = 1.734, RMSEA = .037, GFI = .966, NFI = .981, IFI = .992, SRMR = .042, and PGFI = .653.

*p < .001.

### 4.4 Direct effects of brand perception on experiential satisfaction

As shown in Table 5, all three dimensions of sport event brand perception had significant positive effects on experiential satisfaction. Specifically, brand awareness significantly predicted experiential satisfaction (*β* = .227, *p* < .01), brand image also had a significant positive effect and showed the largest standardized coefficient among the three dimensions (*β* = .358, *p* < .001), and brand trust likewise exerted a significant positive effect on experiential satisfaction (*β* = .334, *p* < .001). Overall, these findings demonstrated that favorable perceptions of the sport event brand significantly enhanced individuals’ experiential satisfaction, thereby supporting the proposed relationship between brand perception and experiential satisfaction.

**Table 5:** Direct effects of the dimensions of brand perception on experiential satisfaction.

Path	*B*	*β*	SE	CR
Brand awareness → Experiential satisfaction	.181	.227	.059	3.089*
Brand image → Experiential satisfaction	.302	.358	.064	4.692**
Brand trust → Experiential satisfaction	.269	.334	.052	5.133**

Model fit indices: *χ*^2^/*df* = 1.469, RMSEA = .030, GFI = .950, NFI = .969, IFI = .990, SRMR = .045, and PGFI = .751.

*p < .01.

**p < .001.

### 4.5 Direct effects of brand perception on emotional connection

As shown in Table 6, all three dimensions of sport event brand perception had significant positive effects on emotional connection. Specifically, brand awareness significantly predicted emotional connection (*β* = .283, *p* < .001), brand image also exerted a significant positive effect (*β* = .226, *p* < .001), and brand trust exerted the strongest positive effect on emotional connection among the three dimensions (*β* = .491, *p* < .001). Overall, these findings indicate that more favorable sport event brand perceptions were associated with stronger emotional connection, thereby supporting the proposed relationship between brand perception and emotional connection.

**Table 6:** Direct effects of the dimensions of brand perception on emotional connection.

Path	*B*	*β*	SE	CR
Brand awareness → Emotional connection	.177	.283	.042	4.232*
Brand image → Emotional connection	.150	.226	.045	3.344*
Brand trust → Emotional connection	.309	.491	.040	7.745*

Model fit indices: *χ*^2^/*df* = 1.384, RMSEA = .027, GFI = .961, NFI = .976, IFI = .993, SRMR = .052, and PGFI = .723.

*p < .001.

### 4.6 Mediating effects of experiential satisfaction and emotional connection

As shown in Table 7, the direct effect of sport event brand perception on revisit intention was not statistically significant (effect =.132, *p* = .963), as both the bias-corrected 95% confidence interval [−5.516, 1.380] and the percentile 95% confidence interval [−3.260, 1.706] included zero. In contrast, the indirect effect through experiential satisfaction was statistically significant (effect =.317, *p* = .010), with both the bias-corrected 95% confidence interval [.087,.683] and the percentile 95% confidence interval [.066,.628] excluding zero, indicating that experiential satisfaction served as a significant mediator between sport event brand perception and revisit intention. However, the indirect effect through emotional connection was not significant (effect =.449, *p* = .225), as both the bias-corrected 95% confidence interval [−.643, 6.350] and the percentile 95% confidence interval [−1.128, 3.874] included zero. Taken together, these findings indicate that experiential satisfaction significantly mediated the relationship between sport event brand perception and revisit intention, whereas emotional connection did not exhibit a significant mediating effect.

**Table 7:** Indirect effects of brand perception on revisit intention through experiential satisfaction and emotional connection.

Path	Effect	SE	Bias-corrected 95% CI		*p*	Percentile 95% CI		*p*
			Lower	Upper		Lower	Upper	
Brand perception → Revisit intention	.132	1.287	−5.516	1.38	.963	−3.26	1.706	.697
Brand perception → Experiential satisfaction → Revisit intention	.317	.180	.087	.683	.010	.066	.628	.016
Brand perception → Emotional connection → Revisit intention	.449	1.27	−.643	6.35	.225	−1.128	3.874	.437

Model fit indices: *χ*^2^/*df* = 1.498, RMSEA = .031, GFI = .916, NFI = .949, IFI = .983, SRMR = .060, and PGFI = .794.

## 5 Discussion

This study examined the relationship between sport event brand perception and revisit intention in the context of the 2024 Beijing Marathon and further tested the mediating roles of experiential satisfaction and emotional connection. The findings provide several important implications.

First, individuals’ overall brand perception of the Beijing Marathon had a significant positive effect on revisit intention. As a higher-order construct reflected by brand awareness, brand image, and brand trust, sport event brand perception represents individuals’ integrated evaluation of the event brand. This finding extends previous sport branding research by demonstrating that overall brand perception remains a robust predictor of revisit intention even in mass-participation endurance events, where individuals directly evaluate event organization, service quality, and experiential value rather than relying primarily on spectator-related symbolic consumption. This finding further suggests that branding plays a broader role in mass-participation sport events by shaping not only pre-event expectations but also individuals’ subsequent evaluations of the overall event experience (57). In large-scale sport events, cognitive image, affective image, and behavioral image also predict revisit intention (47). Expectation-confirmation theory helps explain this pattern. Stable and favorable brand perception can raise pre-event expectations, reduce uncertainty during event selection, and generate more positive evaluations when the actual experience confirms or exceeds those expectations (58). Unlike audiences in traditional spectator sport settings, individuals attending marathon events evaluate multiple aspects of the event throughout their experience, making expectation confirmation a dynamic process that develops before, during, and after the event experience rather than a single post-consumption evaluation (59). In the Beijing Marathon, favorable perceptions of the event brand may have encouraged individuals to form high expectations before attending the event. Notably, brand image appeared to contribute more strongly to overall brand perception than brand awareness and brand trust. This finding may reflect the distinctive characteristics of the Beijing Marathon as one of the most established and prestigious marathon events in China. Compared with brand awareness and brand trust, brand image encompasses broader symbolic meanings and evaluative associations related to event reputation, social recognition, and personal significance (60). In this context, individuals may place greater emphasis on the symbolic and reputational value embodied in the event image, which becomes an important component of their overall evaluation of the Beijing Marathon. Importantly, these findings should be interpreted within the broader context of participatory sport events, where individuals evaluate not only the sporting elements of the event but also organizational quality, service delivery, event atmosphere, and the overall experience generated by the event.

Second, individuals’ brand perception of the Beijing Marathon had a significant positive effect on experiential satisfaction. Rather than merely confirming a positive association, this finding suggests that individuals rely on their overall perceptions of the event brand when interpreting and evaluating the quality of their actual event experience. This result indicates that sport event branding functions not only as a marketing resource for attracting individuals but also as an important cognitive framework through which individuals interpret service quality and experiential value (61). Prior studies have shown that a positive brand image significantly enhances consumer satisfaction, suggesting that favorable perceptions of a product or service lead to more positive evaluations after actual use (62). Research in the sport branding field has also suggested that brand trust develops from reliable products and service quality and can strengthen brand experience through professional, sincere, and responsible service provision, which in turn improves satisfaction (63). Hipólito et al. further showed that trust can reduce consumers’ perceived risk and uncertainty during the service or consumption process, thereby enhancing overall satisfaction with the experience (64). In the Beijing Marathon, favorable perceptions of the event brand may have increased individuals’ familiarity, value perception, and confidence in the event, reduced uncertainty during participation, and ultimately led to more favorable evaluations of the overall event experience. Collectively, these findings further suggest that brand perception shapes not only individuals’ intention to revisit but also the cognitive processes through which event experiences are evaluated.

Third, sport event brand perception had a significant positive effect on emotional connection in the Beijing Marathon context. This result further demonstrates that sport event brand perception influences individuals’ psychological responses through both cognitive evaluation and affective processes, thereby broadening current understanding of the psychological consequences of sport event branding. Previous studies suggest that brand-related perception supports not only consumers’ recognition and memory of a brand but also emotional interaction between the brand and the consumer. High levels of brand-related perception can evoke trust, liking, and belonging, which in turn promote emotional connection (29, 30). Previous studies have further shown that brand trust can provide emotional support in situations characterized by uncertainty and risk and can serve as a psychological basis for maintaining long-term relationships between brands and consumers (65). In addition, a stable and favorable brand image can strengthen emotional attachment and psychological identification, allowing individuals to form closer psychological ties with a brand (66). In the Beijing Marathon, the event's long-standing reputation, favorable image, and reliable organization may have provided individuals with a meaningful object of emotional projection and identification, thereby strengthening emotional attachment during the event experience.

Fourth, experiential satisfaction fully mediated the relationship between sport event brand perception and revisit intention in the Beijing Marathon context. In other words, brand perception did not directly translate into revisit intention; instead, it influenced revisit intention through individuals’ overall evaluation of the event experience. This finding contributes theoretically by demonstrating the full mediating role of experiential satisfaction in a large-scale mass-participation marathon context. Khalil et al. noted that satisfaction not only directly affects revisit intention but also functions as a key mediating or moderating mechanism between situational factors and revisit intention, especially after individuals form positive evaluations of service or experience (67). Other studies have also shown that when the cognitive and affective images of a destination or event improve, individuals’ satisfaction increases significantly, and such satisfaction is then translated into revisit intention (68). In addition, sustainable practices, destination image, perceived value, and novelty seeking have all been found to influence revisit behavior primarily through satisfaction (69). In the Beijing Marathon, favorable brand perception may have improved individuals’ evaluation of the overall event experience and strengthened experiential satisfaction, which in turn increased repeat participation intention. Importantly, the finding of full rather than partial mediation suggests that sport event brand perception alone may not be sufficient to generate revisit intention. Instead, its influence appears to operate primarily through individuals’ evaluations of the actual event experience. This finding highlights the central role of experiential satisfaction in transforming favorable brand-related perceptions into behavioral intentions, suggesting that the value of a strong sport event brand is ultimately realized through the delivery of satisfying event experiences. Taken together, these findings contribute to the sport event branding literature by demonstrating that experiential satisfaction constitutes a key mechanism through which brand value is translated into revisit intention in mass-participation marathon events.

Finally, emotional connection did not significantly mediate the relationship between sport event brand perception and revisit intention in the Beijing Marathon context. Earlier studies have suggested that emotional connection can strengthen individuals’ psychological bonds with brands or destinations and subsequently promote revisit intention; more specifically, emotional attachment can enhance positive brand associations, reinforce trust, and contribute to favorable evaluations and loyalty-related outcomes through memorable experiences (70). However, the present findings suggest that emotional connection may not function as a consistently influential mechanism linking brand-related perceptions and revisit intention across all sport event settings (71). Its influence may vary according to event characteristics and the factors individuals prioritize when evaluating their event experience. Although the estimated indirect effect through emotional connection was positive, the bootstrap confidence intervals included zero, indicating that this mediating pathway was not statistically supported. This result should not be interpreted as evidence that emotional factors are unimportant in marathon participation. Rather, it suggests that emotional connection may play a less central role than experiential satisfaction in shaping individuals’ decisions to revisit the event in this context. This finding challenges the implicit assumption in some sport branding literature that emotional attachment consistently serves as the primary pathway linking brand perception to loyalty-related outcomes. Instead, the present study suggests that evaluations of the actual event experience may play a more decisive role than affective attachment alone when shaping revisit decisions in mass-participation marathon events. Positive feelings toward the event may therefore exist, but they may not be sufficiently central to drive revisit intention once individuals form judgments about the actual event experience (72). Although emotional connection has been identified as an important predictor of loyalty-related outcomes in previous studies, its influence may depend on contextual factors that were not directly examined in the present study. Therefore, the underlying reasons for the non-significant mediating role of emotional connection should be interpreted cautiously and warrant further investigation. Taken together, these findings refine current understanding of how sport event brand perceptions are translated into revisit intention by suggesting that experiential satisfaction represents a more reliable mechanism than emotional connection in mass-participation marathon events.

## 6 Conclusion

This study examined how sport event brand perception shapes revisit intention in the context of the 2024 Beijing Marathon, with particular attention to the mediating roles of experiential satisfaction and emotional connection. The findings indicate that sport event brand perception positively influences experiential satisfaction and emotional connection and that experiential satisfaction serves as the primary mechanism through which brand perception translates into revisit intention. More importantly, experiential satisfaction fully mediates the relationship between brand perception and revisit intention, whereas emotional connection does not exert a significant mediating effect. These results suggest that, in the Beijing Marathon context, revisit decisions depend more on evaluations of the actual event experience than on emotional attachment alone. These findings further suggest that, in highly professional and prestigious mass-participation sport events, individuals place greater emphasis on the quality of event delivery and service performance than on emotional attachment when forming intentions to revisit the event. The study makes three theoretical contributions. First, it clarifies how higher-order sport event brand perception translates into revisit intention by identifying experiential satisfaction as the primary psychological mechanism. Second, it distinguishes the roles of experiential satisfaction and emotional connection, thereby refining sport event branding research. Third, it provides evidence from a large-scale marathon event and highlights the importance of considering event-specific contexts when examining the relative influence of experiential satisfaction and emotional connection on revisit intention. Collectively, these findings contribute to sport event branding research by demonstrating that the mechanisms linking brand perception and revisit intention may differ across sport event contexts. They further highlight the importance of considering the distinctive experiential characteristics of mass-participation sport events when examining revisit behavior.

The findings also offer practical implications for event organizers. Organizers should move beyond image promotion alone and strengthen the service elements that directly shape individuals’ experiences, such as registration communication, race-day information, aid-station efficiency, course support, and post-race recovery. Brand communication should also become more differentiated. For marathon participants, communication strategies should focus on event attributes that enhance the overall participation experience, including course organization, participant support, and race-day services. Improving these aspects may help translate positive brand perceptions into repeat participation intentions. For individuals seeking a stronger sense of identity and belonging, organizers can strengthen emotional storytelling around event heritage, individual achievement, and the symbolic meaning of the host city. At the same time, organizers should recognize that emotional connection is not fostered through symbolic communication alone but is also supported by individuals’ trust in the event brand. Consistent service quality, transparent communication, reliable event operations, and effective responses to individuals’ concerns can strengthen trust in the event brand and, over time, foster deeper psychological and emotional ties between participants and the event. Finally, organizers should establish a systematic post-event feedback mechanism to identify recurring service problems and support continuous improvement across future editions of the event.

This study has several limitations that highlight avenues for future research. First, data were collected from a single event using convenience sampling. Although common in sports event research, this approach limits the overall representativeness of the findings. More importantly, because the sample comprised individuals who voluntarily attended the Beijing Marathon, self-selection bias may be present; these individuals may possess higher baseline enthusiasm, stronger sport involvement, or more favorable pre-existing brand perceptions than the broader sport consumer population. In addition, although the measurement scales were adapted from previously validated instruments, they were designed to capture individuals’ evaluations of the overall event experience rather than race participation alone. Accordingly, the present findings should be interpreted as reflecting individuals’ holistic evaluations of the Beijing Marathon event, and future research is encouraged to develop more role-specific measurement instruments that distinguish runners, spectators, and other participant groups. Second, the cross-sectional research design limits causal inference. It restricts the ability to capture how sport event brand perception, experiential satisfaction, and emotional connection may dynamically evolve over time, particularly given that emotional attachments typically develop gradually through repeated participation. Third, this study focused exclusively on revisit intention. Future research should encompass a broader range of behavioral outcomes relevant to sport branding, such as recommendation intention (word-of-mouth), advocacy behavior, and comprehensive event loyalty. Finally, although the proposed structural model was grounded in established theories, alternative model specifications were not formally compared. Future studies are encouraged to employ longitudinal designs, utilize more representative sampling strategies across multiple international marathon events, and apply multi-group structural equation modeling to test potential moderating effects, such as participant demographics and prior event experience.

## Data Availability

The original contributions presented in the study are included in the article/Supplementary Material, further inquiries can be directed to the corresponding author.

## Appendix A. Measurement Items

**Table:**

Construct	Item
Brand Awareness	I am aware of the Beijing Marathon and familiar with its event brand.
	I frequently hear about or see information regarding the Beijing Marathon.
	I can clearly distinguish the Beijing Marathon brand from other similar marathon events.
Brand Image	The event image of the Beijing Marathon aligns with my travel preferences.
	Watching or participating in the Beijing Marathon reflects my personal interests and values.
	The Beijing Marathon attracts people who share similar interests and lifestyles with me.
	The atmosphere of the Beijing Marathon gives me a sense of enjoyment and belonging.
Brand Trust	I trust the organization and arrangements of the Beijing Marathon event.
	I believe that the organizers of the Beijing Marathon are honest and professional.
	I believe that visiting and participating in the Beijing Marathon is safe and reliable.
Event Quality	Watching top-tier athletes compete makes me feel thrilled and satisfied.
	The athletes outstanding performance enhances my perception of the events professionalism.
	I can conveniently access relevant information about the Beijing Marathon and its participants.
	As a visitor, I can easily find detailed information regarding the Beijing Marathon.
Service Level	The friendly service attitude of the staff makes me feel welcome and at home.
	I enjoy watching the Beijing Marathon alongside visitors and spectators from all over the world.
	Both spectators and participants comply with the event rules, creating a favorable atmosphere.
	The information and assistance provided by volunteers and staff are highly useful to me.
Event Environment	The event atmosphere of the Beijing Marathon allows me to experience the charm of the city.
	I am satisfied with the design of the event venue, including the signage and viewing areas.
	The safety and security of the venue and its surrounding areas give me peace of mind.
	The host location of the marathon event helps me better understand the scenery and culture of Beijing.
Emotional Dependence	The Beijing Marathon holds irreplaceable importance in my travel experience.
	Even when similar events are available, I would still prefer the Beijing Marathon.
	The emotional experience I gain from the Beijing Marathon is difficult to replace with other events.
Brand Identification	The success of the Beijing Marathon gives me a strong sense of pride.
	I would feel disappointed if the Beijing Marathon received negative evaluations.
	I identify with the values delivered by the Beijing Marathon, such as health, vitality, and the spirit of challenge.
Social Connection	Visiting the Beijing Marathon is a great opportunity for me to spend quality time with friends or family.
	I thoroughly enjoy the enthusiastic atmosphere and visitor interactions during the Beijing Marathon event.
	I enjoy discussing event highlights or arrangements with other visitors or spectators.
Revisit Intention	I am willing to participate in activities related to the Beijing Marathon as a visitor again.
	I am willing to include the Beijing Marathon as a part of my travel plans again.
	I will do my best to arrange time to visit or participate in the Beijing Marathon again.
	I am willing to invest time and money to experience the charm of the Beijing Marathon event again.
